# Association of health literacy and medication adherence with health-related quality of life (HRQoL) in patients with ischemic heart disease

**DOI:** 10.1186/s12955-021-01761-5

**Published:** 2021-04-13

**Authors:** Muzna Suhail, Hamid Saeed, Zikria Saleem, Saman Younas, Furqan Khurshid Hashmi, Fawad Rasool, Muhammad Islam, Imran Imran

**Affiliations:** 1 Department of Pharmaceutics, University College of Pharmacy, Universality of the Punjab, Allama Iqbal Campus, Lahore, 54000 Pakistan; 2 Department of Pharmacy, The University of Lahore, Lahore, Pakistan; 3 Department of Pharmacy Practice, Faculty of Pharmacy, Bahauddin Zakariya University, Multan, Pakistan; 4 Department of Pharmacology, Faculty of Pharmacy, Bahauddin Zakariya University, Multan, Pakistan

**Keywords:** Medication adherence, Health literacy, HRQoL, Ischemic heart disease, Lahore, Pakistan

## Abstract

**Background:**

Medication Adherence (MA) and Health Related Quality of Life (HRQoL) are two inter-connected concepts, co-influenced by Health Literacy (HL), with significant impact on patient management and care. Thus, we aimed to estimate the association of HL and MA with HRQoL in IHD patients.

**Methods:**

Cross-sectional study of 251 IHD patients recruited from Lahore over 6 months period. HL, MA and HRQoL was assessed using validated questionnaires; 16-items of HL, Morisky Green Levine Scale (MGLS) and SF-12, respectively. Chi-square for significance, logistic-regression for association and linear regression for predictions were used.

**Results:**

IHD patients; males (*p* = 0.0001), having secondary—higher education (*p* = 0.0001), middle/upper class (*p* = 0.0001) and employed (*p* = 0.005) had adequate HL, and were more likely to be adherent (OR; 4.3, *p* = 0.014). Both physical (PCS-12) and mental (MCS-12) component scores of HRQoL for age, gender, education, area of residence, employment and MA were significantly higher in patients with adequate HL. In multinomial regression, improved PCS-12 scores tend to be higher in subjects having secondary—higher education (OR; 3.5, *p* = 0.067), employed (OR; 6.1, *p* = 0.002) and adherent (OR; 2.95, *p* = 0.218), while MCS-12 scores tend to be higher in patients < 65 years (OR; 2.2, *p* = 0.032), employed (OR; 3, *p* = 0.002) and adherent (OR; 4, *p* = 0.004). In adjusted model, HL (β;0.383, *p* = 0.0001) and MA (β; − 0.133, *p* = 0.018) were significantly associated with PCS-12, and MCS-12 with MA (β; − 0.161, *p* = 0.009) only.

**Conclusion:**

Data suggested that adequate HL was significantly associated with adherence and both physical and mental dimensions of HRQoL were higher in IHD patients with adequate HL. Besides, HL and MA are independent predictors of HRQoL in IHD patients.

## Introduction

Ischemic health disease (IHD), the most common cardiovascular disease (CVD), is the leading cause of morbidity and mortality worldwide [1]. According to one estimate almost 17.5 million people died from CVDs in 2012, representing 31% of all global deaths [2]. Surprisingly, over three quarters of all deaths due to CVD took place in low and middle-income countries [3–5]. Due to complex nature of the disease, the contemporary management of IHD require multifaceted approach—aimed not only on reducing the morbidity and mortality but also improving the quality of life [6, 7].

Health literacy (HL), a cognitive and social skill that enable individuals to access, understand and utilized health information to preserve good health, is a potential risk factor for health outcomes related to physical functioning, such as poor medication adherence, self-management of the disease, health behaviors and reduced utilization of preventive services [8]. Thus, sufficient understanding of health information, disease condition, medication and commitment to prolong therapy is pivotal for IHD patients, and due to chronic nature of the disease they are required to actively participate in the management of their health condition [9, 10]. Nevertheless, for chronic disease management, medications are always the foremost aces, yet, adherence to long-term therapy is persistently poor in such patients [11], which may have severe health related consequences especially for patients with cardiovascular diseases [12]. On an average, for the chronic disease management, almost 50% patients fail to adhere to disease preventive measures, such as taking medication, keeping appointments, screening, exercise and dietary changes [13, 14], which is mainly driven by lack of understanding about the disease and associated medication therapy [15, 16]

Medication adherence (MA), the extent to which a person’s behavior, taking medication, following a diet and life style changes corresponds with agreed recommendations from a health care provider, has been shown to be higher among patients with acute illness compared to those having chronic illness [17, 18]. Several lines of evidences suggest that adherence to cardiovascular medication is sub-optimal in patients with hypertension, coronary heart disease and ischemic heart disease, which continues to undermine their therapeutic efficacy—leading to poor outcomes and higher costs for patients and the health care system [19, 20]

More recently, HRQoL has become an important endpoint in the evaluation of health interventions, particularly in patients with chronic health conditions [21]. HRQoL is defined as the extent to which one’s expected physical, social and emotional well-being is affected by his/her medical condition or the treatment [22]. Literature evidences suggest that both health literacy (HL) and medication adherence (MA), alone or in support, decisively affects health related quality of life (HRQoL) in patient with chronic diseases, such as coronary artery disease, hypertension and diabetes [14, 23–27]. Contrariwise, inadequate health literacy and medication non-adherence can adversely affect the cost of therapy, health outcomes and HRQoL, especially in patients with chronic diseases [23, 27–31]. Several patient related factors affecting patient’s outcomes, such as disease and medication knowledge, lifestyle and behavioral changes, affordability and active self-management of their health conditions, all gambling on HL associated MA, have been shown to impact the HRQoL [24, 32–34]. Seemingly, optimal HL and adherence to medication regimen are critical in improving HRQoL, especially where clinicians struggle to achieve the primary and secondary treatment targets [35].

In Pakistan, where adult literacy stands at 55% (men 67% and women 42%), [14, 36, 37] and majority of the patients with chronic diseases are elderly, having low income, poor education and carry the highest disease burden that may adversely affect the self-care practices, health care usage and disease management [38]. The HRQoL among IHD patients is influenced by several objective and subjective factors that may act together. Keeping in view the above-mentioned existing literature reports, HL, MA and HRQoL may be related and have intricate yet complex relationships that require further investigations—particularly in Pakistani IHD population having poor understanding of the disease and about the treatments. Among South Asian countries, Pakistan has the lowest literacy rate [39] and significantly higher IHD disease burden, though very few literature evidences are available from Pakistan that deal with the impact of HL on HRQoL in patients with ischemic heart disease (IHD) [24], however, not a single study examined the impact of both, HL and MA, significantly associated with each other, on the HRQoL in IHD patients. Thus, the present study was conducted with three purposeful objectives, first the impact of HL on physical and mental components scores of HRQoL, second to estimate the association of HL and HRQoL with patient’s demographics, clinical characteristics and MA and lastly to determine the predictors of physical and mental HRQoL based on HL and MA scores in IHD patients.

## Methodology

### Ethical approval

The present study was approved from the Human Ethical Committee University College of Pharmacy, University of the Punjab, Lahore, reference number HEC/PUCP/1930, and from Institutional Review Board (IRB), King Edward Medical University, Lahore, reference number 320/RC/KEMU. Informed consent was obtained from the participants.

### Study design

A descriptive cross-sectional study was designed to investigate the impact of HL and MA on HRQoL in IHD patients. The study consisted of three distinct portions, i.e. (a) Evaluation of health literacy using a validated 16 items questionnaire developed by chew et al. [40], (b) assessment of MA using Morisky Green Levine Medication Adherence Scale (MGLS) and (c) the impact of both on health-related quality of life (HRQOL) evaluated employing 12-Item Short Form Survey, (SF-12) version 1.0.

### Study settings

Patients were recruited from one of the biggest tertiary care hospital of Lahore, i-e., Mayo hospital (MH) [41], between June 2016–Nov 2016, with influx of patients from all over the Punjab province having estimated population of 110 million [42]. Mayo Hospital was established in 1871 with 2399 beds capacity located in Lahore encomapssing 54.6 acers of land. MH provides, clinical (in-patient & out-patient), diagnostic and emergency services [41]. IHD patients were enrolled from hospital’s out-patient cardiology department.

A comprehensive instrument of measure (questionnaire) was used to collect the data. Questionnaire was administered by field administrator, post graduate pharmacy student, by filling the questionnaire during face-to-face interviews.

### Participants

A total of 251 eligible IHD patients with confirmed clinical diagnosis, as per American College of Cardiology guidelines [43] by a cardiologist, were enrolled in the study from out-patient cardiology department as per study inclusion and exclusion criteria.

*Inclusion criteria* All the eligible IHD patients having confirmed diagnosis as per cardiologist report, above 25 years of age, with disease duration of more than 6 months, irrespective of gender, socio-economic status, ethnicity, the presence of co-morbid conditions and willing to provide informed consent were included in the study.

*Exclusion criteria* All those patients below 25 years of age, disease duration of less than 6 months, terminally ill, cognitive impairment and failed to provide the informed consent were excluded.

Randomization was ensured by giving serial numbers (1, 2, 3….) to all the eligible patients as per clinician’s confirmed IHD diagnosis and later segregating even numbered patients into the study group.

### Variables and data measure

Study variables are categorized into independent and dependent (outcome) variables.

#### Independent variable

Health Literacy was considered an independent variable and was evaluated using a validated 16 item questionnaire developed by chew et al. [40]. Health literacy tool (16-items); a valid questionnaire developed by chew et al. in which every question was rated on a 5-point scale ranging from 0 (always) to 4 (never) except for questions 1–4, 14 and 15 which were reverse coded for calculating health literacy levels, the score ranged from 0–64, which was transformed into categories representing inadequate health literacy (0–34), marginal health literacy (35–42) and adequate health literacy (43–64), but we categorized health literacy as a dichotomous variable between inadequate health literacy (0–34) and adequate health literacy (35–64) by merging the scores of marginal and adequate health literacy [24, 44, 45], only for data analysis and to have very clear and conclusive evidence of the impact of HL, literate (adequate HL) and illiterate (inadequate HL), on both physical and mental HRQoL component scores.

Medication adherence (MA) was also considered as an independent variable for HRQoL but was considered dependent variable in case of HL. MA was assessed using, Morisky Green Levine Scale (MGLS), which is a four-item tool with a suggestive level of MA from high, medium to low with a score ranging from 0 to 4. Morisky Green Levine Scale (MGLS) consisted of 4 items with “yes” and “no” options. The question answered as yes will be scored “1” while the question answered no will be scored “0”. However, MGLS has also been described on the basis of dichotomous definition of adherence, with 1+ points indicating some level of medication non-adherence and 0 indicating perfect level of adherence. Thus, a score between 3–4 (low adherence) and 1–2 (medium adherence) will indicate non-adherence and a score of 0 (high adherence) will indicate MA/perfect adherence [46, 47].

#### Dependent or outcome variable

Health related quality of life (HRQoL) was measured using 12-Item Short Form Survey (SF-12) version 1.0, a non-proprietary version, and the scores were calculated as described previously in original publication [48]. SF-12 (a multipurpose short form) is a shorter alternative to SF-36 and is becoming an instrument of choice to be used in measuring health of both general and specific populations. SF-12 can be administered in two minutes or less and can be either self-administered or through personal interviews [49], SF-12 questionnaire was summarized in to two measures; physical and mental components, which were scored using norm-based methods. Both the PCS-12 (Physical component summary scale-12) and MCS-12 (Mental component summary scale-12) were transformed to contain a mean of 50 and a standard deviation of 10 and all the results were interpreted in accordance with the mean [48].

#### Covariates

Covariates, such as sociodemographic and clinical variables were selected based on the literature reviews [7, 8, 24, 50]. Sociodemographic variables included age (greater or less than 65 years), gender (male or female), marital status (married or unmarried), residence area (rural or urban), education status (no schooling to primary; secondary to higher education), economic status (upper or lower), working status (employed full or part time; unemployed) and clinical variables, such as type of ischemic condition (angina or myocardial infarction), history of revascularization (yes or no), time since first ischemic condition (up to 5 years; more than 5 years), number of other CVD conditions (heart failure, stroke, arrhythmias, cardiomyopathy, heart block, and left ventricular dysfunction) were determined and then converted into ordinal variables (0 and 1 + CVD conditions) and risk factors (Being overweight, diagnosed with hypertension, dyslipidemia, chronic kidney disease and diabetes mellitus) were also determined and later turned into ordinal variables (0–1, 2 and 3+ risk factors). Association of HL and MA was also estimated with co-variates affecting HRQoL.

### Study size

Sample size for the study was determined using Cochran formula [51], taking into account the prevalence of IHD in Pakistan, i-e., 6.25% as of 2009 [52] with 5% precision, 95% confidence interval and infinite population size. The sample size was found to be 91 as per the calculation, yet, 251 eligible IHD patients were enrolled in the study by random selection to compensate the drop outs due to missing data.

### Statistical methods

Chi-square test was used to determine frequency distribution of categorical variables with respect to health literacy. Confidence intervals of 95% were also determined. The differences in the mean scores of physical and mental components of Health-related quality of life among sociodemographic and clinical variables was determined by using independent t-test.

Descriptive statistics was used to determine the mean of physical and mental components of quality of life. Binary and multinomial regression were used to measure the associations. Linear regression model was used to evaluate β-regression coefficients, crude and adjusted, to determine the possible predictors of HRQoL. P-value of < 0.05 was used as an indicator of statistical significance. Data were analyzed using IBM SPSS Statistics Version 20.

## Results

### Demographics and clinical characteristics of IHD patients according to health literacy level

Patient’s demographics and clinical characteristics as per health literacy [52] levels, adequate HL (**HL**_**AD**_) and inadequate HL (**HL**_**IAD**_), are summarized in Table 1. Data revealed that majority of the patients (> 70%) were < 65 years of age with no difference in the frequency distribution of adequate and inadequate HL levels, though scores were markedly different in both age groups based on which HL levels were defined, adequate and inadequate (***scores;*** < 65: **HL**_**IAD;**_ 11.98 ± 10.44, **HL**_**AD;**_ 48.71 ± 8.89, > 65: **HL**_**IAD;**_ 11.48 ± 10.74, **HL**_**AD;**_ 49.18 ± 9.61). Compared to females, males (**HL**_**AD**_**;** 78.7%, **HL**_**IAD**_**;** 55.7%, *p* = 0.001) had adequate HL. Patients from middle class (50.7%) families (***scores: HL***_**IAD;**_ 19.72 ± 10.69, **HL**_**AD;**_ 50.47 ± 8.89) and those having secondary—higher secondary education (74.7%, *p* = 0.0001, ***scores: HL***_**IAD;**_ 29.37 ± 5.60, **HL**_**AD;**_ 50.27 ± 9.14) had adequate HL, while patients belonging to lower class (83.5%, p = 0.001, ***scores: HL***_**IAD;**_ 10.2 ± 9.57, **HL**_**AD;**_ 46.32 ± 8.41) families had inadequate HL. Moreover, the frequency distribution regarding employment status (*p* = 0.005), majority unemployed (***scores: HL***_**IAD;**_ 11.37 ± 10.22, **HL**_**AD;**_ 48.15 ± 9.08), and history of vascularization (*p* = 0.0001, ***scores: HL***_**IAD;**_ 14.05 ± 12.02, **HL**_**AD;**_ 49.61 ± 8.38) differed significantly with HL level. However, HL levels were not significantly different with regards to area of residence (*p* = 0.682), types of ischemic condition (*p* = 0.619), no. of other CVD conditions (*p* = 0.891), no. of clinical risk factors (*p* = 0.724) and MA (*p* = 0.229) (Table 1).

**Table 1:** Demographic clinical characteristics of Ischemic heart disease (IHD) patient according to health literacy

Characteristics	Health literacy, *n* = 251				*p *values
	Inadequate, *n* = 176		Adequate, *n* = 75		
	Frequency (%)	Average score ± SD	Frequency (%)	Average score ± SD	
*Age (years)*					
< 65	128 (72.7)	11.98 ± 10.44	58 (77.3)	48.71 ± 8.89	0.446
> 65	48 (27.3)	11.48 ± 10.74	17 (22.7)	49.18 ± 9.61	
*Gender*					
Male	98 (55.7)	14.90 ± 11.32	59 (78.7)	49.22 ± 8.77	0.001**
Female	78 (44.3)	8.01 ± 7.88	16 (21.3)	47.31 ± 9.93	
*Residence area*					
Urban	154 (87.5)	12.39 ± 10.64	67 (89.3)	49.27 ± 9.07	0.682
Rural	22 (12.5)	8.05 ± 8.66	8 (10.7)	45.00 ± 7.82	
*Educational status*					
No—primary education	160 (90.9)	10.09 ± 9.178	19 (25.3)	44.53 ± 7.14	0.0001**
Secondary—higher education	16 (19.1)	29.37 ± 5.60	56 (74.7)	50.27 ± 9.14	
*Social status*					
Upper	4 (2.3)	23 ± 15.47	6 (8)	51.17 ± 10.98	0.0001**
Middle	25 (14.2)	19.72 ± 10.69	38 (50.7)	50.47 ± 8.89	
Lower	147 (83.5)	10.2 ± 9.57	31 (41.3)	46.32 ± 8.41	
*Employment status*					
Employed full or part time	34 (19.3)	15.38 ± 11.00	27 (36.0)	50.00 ± 8.88	0.005*
Unemployed	142 (80.7)	11.00 ± 10.22	48 (64.0)	48.15 ± 9.08	
*Type of ischemic condition*					
Angina	35 (19.9)	12.34 ± 10.11	17 (22.7)	45.00 ± 8.53	0.619
Myocardial Infarction	141 (80.1)	11.72 ± 10.62	58 (77.3)	49.93 ± 8.88	
*History of revascularization*					
Yes	43 (24.4)	14.05 ± 12.02	38 (50.7)	49.61 ± 8.38	0.0001**
No	133 (75.6)	11.14 ± 9.90	37 (49.3)	48.00 ± 9.63	
*Time since first ischemic episodes (years)*					
≤ 5	135 (76.7)	12.30 ± 10.6	57 (76.0)	49.09 ± 8.84	0.904
> 5	41 (23.3)	10.34 ± 10.10	18 (24.0)	47.94 ± 9.67	
*Number of other CVD conditions*					
0	161 (91.5)	11.98 ± 10.59	69 (92.0)	49.45 ± 9.04	0.891
≥ 1	15 (8.5)	10.40 ± 9.58	6 (8.0)	41.50 ± 4.04	
*Number of CVD risk factors*					
0–1	58 (33.0)	15.76 ± 11.61	24 (32.0)	48.75 ± 8.86	
2	57 (32.4)	9.65 ± 9.43	28 (37.3)	48.14 ± 8.53	0.724
≥ 3	61 (34.7)	10.18 ± 9.37	23 (30.7)	49.70 ± 9.97	
*Medication adherence*					
Adherent	136 (77.3)	12.16 ± 10.14	63 (84)	49.06 ± 8.91	0.229
Non-adherent	40 (22.7)	10.78 ± 11.68	12 (16)	47.50 ± 9.72	

*p* values were estimated based on differences in frequency distribution

CVD = cardiovascular disease. Health Literacy (HL)Scores; Inadequate = 0–34, Adequate = 35–64, SD; standard deviation

*p ≤ 0.05–0.002, **≤ 0.001–0.0001

### Physical and mental component summary of HRQoL in IHD patients as per HL level

The physical (PCS-12) and mental (MCS-12) component summaries of HRQoL in IHD patients as per health literacy levels are summarized in Table 2. Data suggested that PCS-12 scores were significantly different with regards to HL for age < 65 (*p* = 0.001) and > 65 (*p* = 0.001) years, gender; male (*p* = 0.001) and female (*p* = 0.008), area of residence; urban (*p* = 0.001) and rural (*p* = 0.001), primary education (*p* = 0.045), socioeconomic status; upper (*p* = 0.026) and lower (*p* = 0.001), employment status; employed (*p* = 0.001) and un-employed (*p* = 0.001), history of re-vascularization, yes (*p* = 0.001) and no (*p* = 0.001), other CVD conditions; 0 (*p* = 0.001) and ≥ 1 (*p* = 0.008), clinical CVD risk factors (*p* < 0.005) and MA; adequate (*p* = 0.023) (Table 2).

**Table 2:** Physical and Mental component summary of HRQoL in IHD Patients according to Health Literacy Levels

Variables	Health related quality of life (*HRQoL*) components					
	PCS-12 (mean ± SD)			MCS-12 (mean ± SD)		
	Health literacy					
	Inadequate	Adequate	*p* value	Inadequate	Adequate	*p* value
*Age*						
< 65 years	26.53 ± 8.64	37.62 ± 12.54	0.001**	39.47 ± 14.99	49.06 ± 11.97	0.001**
> 65 years	27.46 ± 7.90	39.83 ± 10.47	0.001**	42.17 ± 13.75	47.00 ± 10.37	0.26
*Gender*						
Male	28.46 ± 9.76	37.62 ± 11.97	0.001**	42.30 ± 12.67	49.32 ± 11.25	0.002*
Female	24.68 ± 5.78	40.92 ± 12.65	0.008*	37.57 ± 16.57	44.61 ± 12.99	0.248
*Residence area*						
Urban	27.02 ± 8.49	37.80 ± 12.50	0.001**	41.16 ± 14.97	48.51 ± 11.85	0.001**
Rural	25.16 ± 7.97	41.26 ± 5.90	0.001**	33.52 ± 10.39	49.07 ± 9.26	0.014*
*Educational status*		36.68 ± 13.48	0.045*	39.79 ± 15.03	51.19 ± 10.20	0.01*
No—primary education	25.97 ± 8.28	38.47 ± 11.83	0.263	44.34 ± 9.99	47.99 ± 11.85	0.281
Secondary—higher education	35.00 ± 4.84					
*Socioeconomic status*						
Upper	30.13 ± 8.27	45.87 ± 6.59	0.026*	42.83 ± 0.52	49.75 ± 9.27	0.026*
Middle	31.05 ± 9.97	37.07 ± 11.91	0.055	45.54 ± 11.99	47.74 ± 12.64	0.523
Lower	25.97 ± 7.96	38.05 ± 12.87	0.001**	39.22 ± 15.12	49.49 ± 10.73	0.001**
*Employment status*						
Employed	30.79 ± 10.22	41.10 ± 12.56	0.001**	45.36 ± 14.09	50.71 ± 11.73	0.111
Un-employed	25.83 ± 7.68	33.47 ± 9.97	0.001**	38.97 ± 14.59	33.24 ± 9.98	0.016*
*Type of Ischemic condition*						
Angina	28.67 ± 10.50	38.24 ± 12.86	0.081	40.49 ± 13.17	48.47 ± 10.93	0.120
Myocardial infarction	26.32 ± 7.81	38.13 ± 12.01	0.001**	40.13 ± 15.07	48.59 ± 11.78	0.001**
*History of revascularization*						
Yes	27.87 ± 8.09	37.21 ± 12.45	0.001**	45.83 ± 12.81	49.61 ± 11.09	0.200
No	26.44 ± 8.54	39.44 ± 11.56	0.001**	38.38 ± 14.82	47.13 ± 12.26	0.006*
*Time since first ischemic episode (years)*						
≤ 5	27.42 ± 9.34	37.66 ± 13.15	0.001**	41.42 ± 15.06	48.66 ± 11.41	0.002*
> 5	25.5 ± 6.38	39.87 ± 6.76	0.001**	36.89 ± 12.52	48.24 ± 12.55	0.017*
*Other CVD conditions*						
0	26.88 ± 8.65	38.13 ± 12.37	0.001**	40.73 ± 12.86	48.91 ± 11.80	0.001**
≥ 1	20.01 ± 5.72	38.43 ± 3.77	0.008*	35.64 ± 28.38	43.25 ± 3.77	0.658
*Clinical CVD risk factors*						
0–1	28.47 ± 8.86	39.99 ± 13.44	0.004*	42.45 ± 12.36	43.57 ± 11.65	0.747
2	26.52 ± 9.08	37.43 ± 10.88	0.001**	40.49 ± 17.35	52.00 ± 10.47	0.001**
≥ 3	25.43 ± 7.14	37.08 ± 12.39	0.003*	37.79 ± 13.84	49.56 ± 11.57	0.002*
*Medication adherence*						
Adherent	25.99 ± 10.5	34.91 ± 11.31	0.0235*	42.29 ± 14.88	51.3 ± 10.12	0.0053*
Non-adherent	–	27.36 ± 8.71	–	–	38.12 ± 11.69	–

*SD *standard deviation, *CVD *ardiovascular disease

*p ≤ 0.05–0.002, **≤ 0.001–0.0001

Likewise, the MCS-12 scores were significantly different with regards to HL for area of residence; urban (*p* = 0.001) and rural (*p* = 0.014), no or primary education (*p* = 0.01), socio-economic status; lower (*p* = 0.001) and upper (*p* = 0.026), and MA; adherent (*p* = 0.0053). While contrary to PCS-12 scores, MCS-12 scores exhibited differences with regards to HL only for patients < 65 years (*p* = 0.001) of age, male gender (*p* = 0.002), un-employed status (*p* = 0.016), no history of re-vascularization (*p* = 0.006), no other CVD condition (*p* = 0.001) and clinical CVD risk factors of 2 (*p* = 0.001) or > 3 (*p* = 0.002) (Table 2).

### Association of health literacy with patient’s demographics, clinical characteristics and MA

Data regarding the association of HL with demographics, clinical characteristics and MA in IHD patients are summarized in Table 3. As shown in Table 3., results of binary regression suggested that adequate literacy was significantly associated with secondary to higher education (OR; 19.3, *p* = 0.0001), no history of re-vascularization (OR; 0.28, *p* = 0.0001) and adequate MA (OR; 4.3, *p* = 0.014). Besides, HL demonstrated no significant association with rest of the variables; demographic and clinical (Table 3).

**Table 3:** Association of Health literacy with demographics, clinical characteristics and medication adherence in IHD patients

Characteristics	Health literacy		Binary logistic	*p *values
	Adequate, *n* = 75	Inadequate, *n* = 176	OR (CI)	
*Age*				
< 65 (reference)	58	128	0.8 (0.33–2.1)	0.708
> 65	17	48		
*Gender*				
Male	59	98	1.3 (0.54–2.9)	0.59
Female (reference)	16	78		
*Area of residence*				
Urban (reference)	67	154	1.18 (0.27–4.1)	0.962
Rural	8	22		
*Education*				
No—primary (reference)	19	160	19.3 (8.3–45.1)	0.0001**
Secondary—higher	56	16		
*Employment status*				
Employed	27	34	1.94 (0.82–4.6)	0.132
Unemployed (reference)	48	142		
*Socioeconomic status*				
Lower (reference)	8	4		
Middle	38	25	2 (0.35–11.8)	0.43
Upper	31	147	2.1 (0.83–5.3)	0.113
*Time since first episode (years)*				
≤ 5 (reference)	57	135	1.2 (0.58–2.3)	0.69
> 5	18	41		
*History of re-vascularization*				
Yes (reference)	38	43	0.28 (0.15–0.51)	0.0001**
No	37	133		
*Type of ischemic condition*				
Angina	17	35	1.61 (0.78–3.3)	0.198
Myocardial infarction (reference)	58	141		
*No. of CVD risk factors*				
0–1 (reference)	24	58	2.1 (0.81–5.78)	0.101
2	28	57	1.3 (0.48–3.6)	0.534
≥ 3	23	61		
*Medication adherence*				
Adherent	63	136	4.3 (1.34–13.7)	0.014*
Non-adherent (reference)	12	40		

*OR* odds ratio, *CVD* cardiovascular disease, *CI* confidence interval

*p ≤ 0.05–0.002; **≤ 0.001–0.0001

### Association of HRQoL, PCS-12 and MCS-12, with patient’s demographics, clinical characteristics and MA

Binary regression data presented in Table 4, revealed that IHD patients who were employed were more likely to exhibit improved PCS-12 scores (OR; 6.4, *p* = 0.004), while no significant association was observed between PCS-12 and rest of the variables, nevertheless, IHD patients with adequate MA (OR; 3.2, *p* = 0.225) were more likely to demonstrate improved PCS-12 scores.

**Table 4:** Association of HRQoL, physical component summary (PCS-12) and mental component summary (MCS-12), with demographics, clinical characteristics and medication adherence in IHD patients

Characteristics	PCS-12, *n* = 251			*p *values	MCS-12, *n* = 251			*p *values
	Improving (≥ 50), *n* = 19	Not improving (< 50) *n* = 232	Binary logistic		Improving (≥ 50), *n* = 82	Not improving (< 50) *n* = 169	Binary logistic	
			OR (CI)				OR (CI)	
*Age*			1.1 (0.34–18.4)	0.91			2.2 (1.1–4.94)	0.034*
< 65	16	170			57	129		
> 65	3	62			25	40		
*Gender*			1.6 (0.37–6.7)	0.544			1.7 (0.87–3.3)	0.120
Male	16	141			61	96		
Female	3	91			21	73		
*Education*								
No—primary	8	171			51	128		
Secondary—higher	11	61	3.5 (0.93–13.1)	0.063	31	41	1.6 (0.75–3.7)	0.208
*Employment status*								
Employed	12	49	6.4 (1.8–22.5)	0.004*	32	29	3 (1.5–6.2)	0.002*
Unemployed	7	183			50	140		
*Time since first episode (years)*							0.59 (0.28–1.3)	0.184
≤ 5	18	174	0.14 (0.016–1.25)	0.079	68	124		
> 5	1	58			14	45		
*History of re-vascularization*								
Yes	7	74	0.69 (0.19–2.5)	0.567	36	45	1.72 (0.91–3.3)	0.098
No	12	158			46	124		
*Type of ischemic condition*			1.8 (0.46–7.1)	0.402			0.61 (0.27–1.4)	0.221
Angina	5	47			18	34		
Myocardial infarction	14	185			64	135		
*No. of CVD risk factors*								
0–1	9	73	1.4 (0.38–5.2)	0.607	26	56	0.83 (0.38–1.8)	0.639
2	5	80	0.77 (0.18–3.24)	0.717	32	53	1.6 (0.73–3.3)	0.256
≥ 3	5	79			24	60		
*Medication adherence*			3.2 (0.49–20.1)	0.225			4 (1.6–10.3)	0.004*
Adherent	17	182			74	125		
Non-adherent	2	50			8	44		

*OR* odds ratio, *CVD* cardiovascular disease, *CI* confidence interval

*p ≤ 0.05–0.002, **≤ 0.001–0.0001

Moreover, HRQoL, mental component summary (MCS-12) association data suggested that IHD patients < 65 years of age (OR; 2.2, *p* = 0.034), employment status (OR; 3, *p* = 0.002) and adequate MA (OR; 4, *p* = 0.004) were more likely to demonstrate improved MCS scores. While, no significant associations were observed for rest of the variables, nonetheless, the secondary to higher education (OR; 1.6, *p* = 0.208) and no history of re-vascularization (OR; 1.72, *p* = 0.098) were more likely to have improved MCS-12 scores (Table 4).

### Predictors of HRQoL

Data regarding predictors of HRQoL, PCS-12 and MCS-12, in IHD patients are summarized in Table 5. In both un-adjusted and adjusted model, HL scores (*un-adjusted:* β; 0.513, *p* = 0.0001, *adjusted:* β; 0.383, *p* = 0.0001), literacy levels, adequate (*un-adjusted:* β; -0.412, *p* = 0.0001, *adjusted:* β; -0.196, *p* = 0.005) and in-adequate (*un-adjusted:* β; 0.417, *p* = 0.0001, *adjusted:* β; 0.208, *p* = 0.005) were significantly associated with physical (PCS-12) HRQoL, while the MA scores *(adjusted:* β; -0.133, *p* = 0.018) and levels of MA, high *(adjusted:* β; 0.124, *p* = 0.027) and low *(adjusted:* β; 0.134, *p* = 0.018) were significantly associated with PCS-12 only in the adjusted model (Table 5).

**Table 5:** Predictors of HRQoL in IHD patients

Characteristics	PCS-12			
	Unadjusted		Adjusted^ǂ^	
	β-coefficients (CI)	*p* values	β-coefficients (CI)	*p *values
Health literacy score	0.513 (0.21 to 0.33)	0.0001**	0.383 (0.12 to 0.29)	0.0001**
Adequate	− 0.412 (− 13.6 to − 7.7)	0.0001**	− 0.196 (− 8.6 to − 1.5)	0.005*
Marginal	− 0.08 (− 7.3 to 1.2)	0.163	− 0.016 (− 4.5 to 3.4)	0.783
Inadequate	0.417 (6.9 to 11.9)	0.0001**	0.208 (1.4 to 7.9)	0.005*
Medication adherence score	− 0.123 (− 1.8 to 0.008)	0.052	− 0.133 (− 1.7 to − 0.17)	0.018*
High	− 0.095 (− 4.6 to 0.59)	0.132	− 0.124 (− 4.9 to − 0.29)	0.027*
Medium	0.006 (− 2.6 to 2.9)	0.924	0.020 (− 2 to 2.9)	0.725
Low	0.110 (− 0.35 to 5.9)	0.081	0.134 (0.58 to 6.3)	0.018*
Time since first episode (years)	− 0.042 (− 4.1 to 2)	0.510	− 0.052 (− 3.9 to 1.4)	0.356
History of re-vascularization	− 0.115 (− 5.3 to 0.195)	0.069	− 0.008 (− 2.7 to 2.3)	0.889
Type of ischemic condition	− 0.076 (− 5.1 to 1.2)	0.230	− 0.076 (− 4.9 to 0.97)	0.190
No. of CVD risk factors	− 0.136 (− 3.3 to − 0.16)	0.031*	− 0.091 (− 2.6 to 0.252)	0.107

Characteristics	MCS− 12			
	Unadjusted		Adjusted^ǂ^	
	β-coefficients (CI)	*p* values	β-coefficients (CI)	*p *values
Health literacy score	0.241 (0.086 to 0.261)	0.001**	0.107 (− 0.053 to 0.207)	0.242
Adequate	− 0.224 (− 12.24 to − 3.6)	0.0001**	− 0.108 (− 9.1 to 1.53)	0.16
Marginal	0.103 (− 1.8 to 11.6)	0.151	0.049 (− 4.6 to 9.1)	0.505
Inadequate	0.270 (3.9 to 12.7)	0.0001**	0.147 (− 1.2 to 10.3)	0.119
Medication adherence score	− 0.175 (− 2.9 to − 0.51)	0.005*	− 0.161 (− 2.7 to − 0.4)	0.009*
High	− 0.147 (− 7.7 to − 0.68)	0.019*	− 0.163 (− 8 to − 1.3)	0.007*
Medium	0.027 (− 2.9 to 4.6)	0.676	0.078 (− 1.8 to 5.5)	0.329
Low	0.151 (0.97 to 9.6)	0.017*	0.136 (0.52 to 9)	0.028*
Time since first episode (*Yrs*)	− 0.096 (− 7.4 to 0.93)	0.127	− 0.107 (− 7.6 to 0.43)	0.080
History of re-vascularization	− 0.225 (− 10.5 to − 3.1)	0.0001**	− 0.162 (− 8.6 to − 1.2)	0.009*
Type of ischemic condition	− 0.009 (− 4.7 to 4)	0.886	− 0.046 (− 5.9 to 2.7)	0.469
No. of CVD risk factors	− 0.070 (− 3.4 to 0.95)	0.268	− 0.033 (− 2.7 to 1.5)	0.587

*CVD* cardiovascular disease, *PCS* physical component summary, *MCS* mental component summary

*p ≤ 0.05–0.002, **≤ 0.001–0.0001, **ǂ** Adjusted for Co-variates; age, gender, marital status, education, area of residence, employment status

Furthermore, mental (MCS-12) HRQoL was significantly associated with HL scores *(adjusted:* β; 0.241, *p* = 0.0001) and level of HL, adequate *(adjusted:* β; -0.224, *p* = 0.0001) and inadequate *(adjusted:* β; 0.270, *p* = 0.0001) in un-adjusted model only. Contrary to physical HRQoL, MCS-12 was significantly associated with MA scores (*un-adjusted:* β; -0.175, *p* = 0.005, *adjusted:* β; -0.161, *p* = 0.009) and level of MA, high (*un-adjusted:* β; -0.147, *p* = 0.019, *adjusted:* β; -0.163, *p* = 0.007) and low (*un-adjusted:* β; 0.151, *p* = 0.017, *adjusted:* β; 0.136, *p* = 0.028) in both un-adjusted and adjusted models. Besides, only MCS-12 was significantly associated with history of re-vascularization (*un-adjusted:* β; -0.225, *p* = 0.0001, *adjusted:* β; -0.162, *p* = 0.009) in both unadjusted and adjusted models (Table 5).

No significant associations were observed for rest of the variables with either physical or mental HRQoL (Table 5).

## Discussion

Numerous literature reports suggest that health literacy can impact the quality of life of CVD patients, yet most of these have been limited to the patients with heart failure [35, 53]. The intricate relationship between HL and HRQoL is explained by insolvent adherence to life style changes and pharmacological treatments among patients with inadequate health literacy affecting HRQoL [28, 35]. Yet the association of HL and HRQoL with IHD patient’s demographics, clinical characteristics and MA remain unclear. In the present study, the first from Pakistan, we observed that in IHD patients HL literacy was significantly different with regards to age, education, employment status, social class and history of re-vascularization. Likewise, based on HL, physical and mental health components of HRQoL were significantly different in IHD patients < 65 years of age, no or primary education, upper and lower social class, un-employed, no history of re-vascularization, 2 or more clinical CVD risk factors and adequate MA. Additionally, HRQoL, PCS-12, was significantly associated with HL, adequate and in-adequate levels, MA score, high and poor adherence levels, even when adjusted for co-variates. While, MCS-12 was significantly associated with HL score and HL levels in un-adjusted model only, however, MA score, high and low adherence levels and history of re-vascularization demonstrated significant association with MCS-12 even when adjusted for co-variates.

During the past decades, besides traditional end points that may not depict the impact of interventions on patient’s HRQoL, there has been growing concern and interest in assessing the patient’s HRQoL, especially in patients with chronic diseases [54]. In this context, the chronic nature of IHD requires the patient to adequately understand the disease and therapy in order to actively participate in the management of his/her health condition. We observed that only 30% IHD patients exhibited adequate HL, out of which majority of them were males, had secondary/higher secondary education, hailing from the middle class, employed and were adherent to their medication. Albeit, no study was available from Pakistan on IHD patients for direct comparison, yet a study on elderly hypertensive patients of Islamabad, federal capital city, reported adequate HL in 37.4% subjects [55]. We have reported previously that HL was 33% among diabetic patients, more closer to the findings of the current study, with higher frequency of adequate HL in middle class, well-educated and employed subjects [14]. The differences in the HL levels reported in our studies and the one conducted in Islamabad on elderly hypertensive patients may be attributed to the differences in the HL assessment tools, more complex disease condition, i-e., IHD, and more educated working-class residents in Islamabad with white collar jobs compared to Lahore. Additionally, similar to our findings, studies from Taiwan [56], Serbia [57] and Spain [58] reported adequate HL of 36%, 38.9% and 21%, respectively, among CVD patients.

Moreover, HL is greatly associated with MA [59], since inadequate HL leads to weak or almost no MA [60]. We observed that 47.4% of the IHD patients had adequate MA, which was significantly associated with HL. Two studies from federal capital city, Islamabad, though direct comparison cannot be made, reported adequate MA of 38.9% [55] and 38.3% [61] in hypertensive patients, while one study demonstrated significant association of HL with adequate MA [55]—corroborating our findings, yet in different disease condition. It has been assumed that the presence of co-morbid conditions, quite common in IHD patients, modulate the attitude of such patients towards a stricter medication-taking behavior [62], which could be one reason of the improved MA in IHD patients compared to hypertensive population of Islamabad.

Several lines of literature evidences suggest considerable association of HRQoL with HL [35, 53] and MA in patients with chronic diseases [63]. Yet, only a few studies evaluated the impact of HL and MA on IHD patient’s HRQoL, not a single from Pakistan. Our data suggested that both physical and mental component summary scores, segregated based on demographic and clinical parameters, were significantly higher in subjects with adequate HL—though inadequate HL exhibited stronger effect on physical component of HRQoL in IHD patients as reported previously for patients with chronic diseases [24, 64]. Besides, no significant associations were observed for both physical and mental components of HRQoL with patient characteristics except for employment status—suggesting that the patient’s ability to go for a job and earn would have a positive impact on HRQoL, particularly relating to the emotional dimension of HRQoL. We further observed that MCS-12 was significantly associated with adequate MA, a finding that is in agreement with previously published reports that MA is significantly associated with mental component of HRQoL in patients with pulmonary tuberculosis [65] and older hypertensive patients [66]. Data further suggested that in IHD patients, health literacy was an independent predictor of HRQoL, physical and mental components, however, when adjusted for co-variates, HL still remained an independent predictor of physical dimension of HRQoL, corroborated by previous report in heart failure patients [57]. MA and HRQoL are important indicators of therapeutic success, while adherence to therapy drives changes in HRQoL [67]. We observed that MA was significantly associated with emotional dimension of HRQoL, un-adjusted and adjusted, but its association with physical dimension of HRQoL was observed only when adjusted for co-variates. These data clearly demonstrated that HL and MA are independent predictors of HRQoL, though with variables impact on physical and emotional dimensions of HRQoL.

### Study clinical implication and policy recommendations

In the hierarchical cluster of chronic disease management, understanding about the disease and therapy, i.e., HL lies on the top, the effect of which trickles down to any linked actions on patient’s side, such self-care and management in form of adherence to therapy and life style modifications having far reaching impact on patient’s HRQoL. We and others have shown previously that in developing countries, including Pakistan, where majority have poor finances and limited access to education, HL is an unexplored entity, created and refined by the developed world—though people in Pakistan are conscientiously oblivious of the term HRQoL. Unlike, diabetes, IHD patients require more intricate self-care and management stratagems that implicitly and exclusively demand minimal disease and therapy related understanding to practice optimal self-care and management skills having significant impact on HRQoL. Therefore, much responsibility lies on health professionals in refining HL in IHD patients, which is not only associated to MA but in turn would improve HRQoL in IHD patients.

In this regard, integrative patient-centered approach by a team of skilled health professionals, doctors, pharmacists and nurses, can play pivotal role in improving HRQoL in IHD patients. As HRQoL cannot be extrapolated from routine clinical variables, therefore, in IHD patients, HRQoL should specifically be assessed in routine clinical examination to better treat the non-physical aspects of the disease having direct bearing on the management and the promotion of HRQoL in IHD patients. Besides, as observed in our study, HL is significantly associated with MA and HRQoL in IHD patients, by achieving marginal to adequate HL levels would immanently improve both the MA and HRQoL in these patients. Thus, clinical pharmacist services should be mandated to educate and counsel IHD patients to achieve these non-clinical, yet pivotal targets with upfront influence on HRQoL.

### Study limitations

Our study has a few limitations. The cross-sectional design of the study did not permit to observer patients over a period of time and interpret causal relationship between the variables. Also, the small sample size (n = 251) would limit the generalizability of the study findings. Moreover, face to face interviews were conducted for data collection that may lead to reporting bias due to the influence of social desirability factor, which might compel the participants to give desirable answers related to their quality of life to the interviewer. Finally, direct comparison cannot be done due to non-availability of any similar study in IHD patients from Pakistan.

## Conclusions

In conclusion, our results suggest that IHD patients having inadequate HL have significantly reduced physical and psychological dimensions of HRQoL, but with more significant impact on physical functioning. Besides, adherent IHD patients had adequate HL and improved physical and psychological dimensions of HRQoL. Data further suggested that HL and MA are independent predictors of HRQoL in IHD patients. Therefore, during every consultation with IHD patients, health professionals, doctors and pharmacists, should consider inadequate HL as a potential barrier to MA and provide effective care that has significant impact on health outcomes with subsequent effects on physical dimensions of HRQoL.

## Data Availability

Can be obtained from corresponding author upon a reasonable request.

## Abbreviations

HL: Health literacy
MA: Medication adherence
HRQoL: Health related quality of life
IHD: Ischemic heart disease
MGLS: Morisky Green Levine Scale
SF-12: 12-Item Short Form Survey
BR: Binary regression
MR: Multinomial regression
HL_AD_: Adequate health literacy
HL_IAD_: Inadequate health literacy
PCS-12: Physical component summary-12
MCS-12: Mental component summary-12
